# Supplementary figures and images for: Simulating international tax designs on sugar-sweetened beverages in Mexico
Source: PLoS One. 2021 Aug 19;16(8):e0253748. doi: 10.1371/journal.pone.0253748 (PMC8375996; doi:10.1371/journal.pone.0253748)

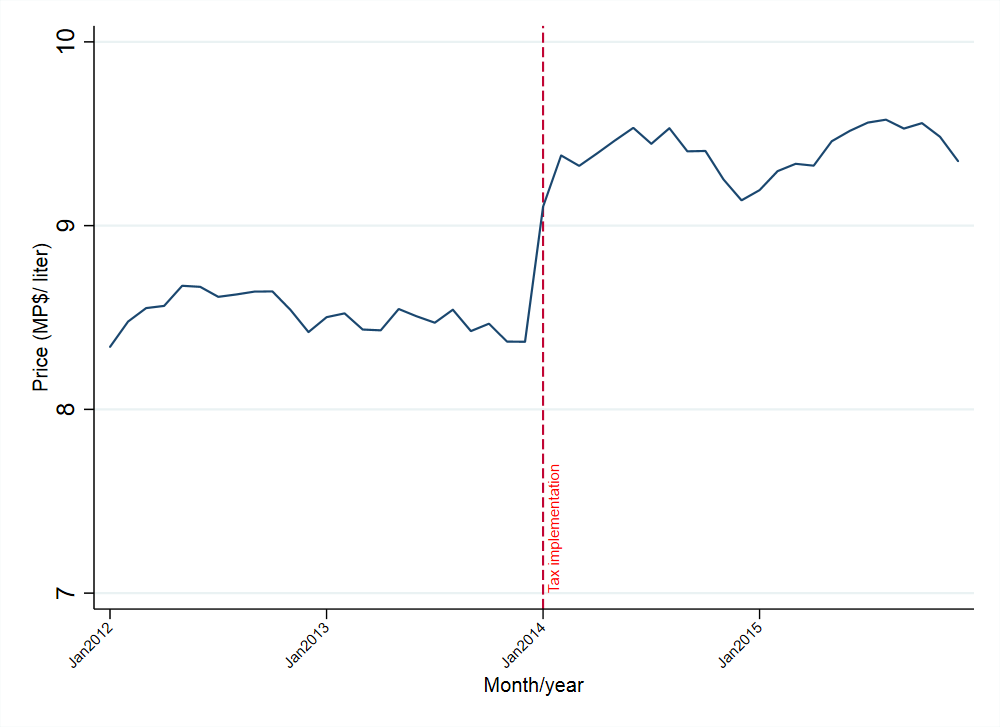

Supplement: S1 Fig — Note: Prices are calculated as quantity-weighted average prices. Source: Authors’ own analyses and calculations based on data from Nielsen through its Mexico Consumer Panel Service (CPS) for the food and beverage categories for January 2012 –December 2015. The Nielsen Company, 2016. Nielsen is not responsible for and had no role in preparing the results reported herein. (TIF) [file pone.0253748.s001.tif]

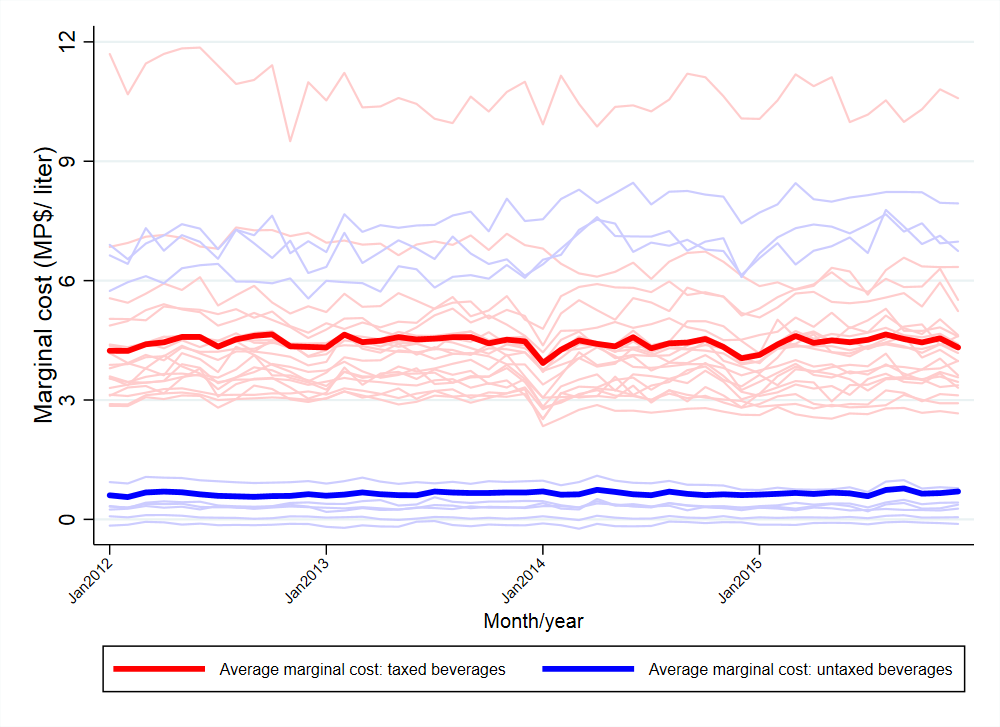

Supplement: S2 Fig — Note: Average marginal costs for the full categories of taxed and untaxed beverages calculated as quantity-weighted average marginal costs. Light red (blue) lines represent the marginal costs for individual taxed (untaxed) products. Source: Authors’ own analyses and calculations based on data from Nielsen through its Mexico Consumer Panel Service (CPS) for the food and beverage categories for January 2012 –December 2015. The Nielsen Company, 2016. Nielsen is not responsible for and had no role in preparing the results reported herein. (TIF) [file pone.0253748.s002.tif]
