# Supplementary material for: Simulating international tax designs on sugar-sweetened beverages in Mexico
Source: PLoS One. 2021 Aug 19;16(8):e0253748. doi: 10.1371/journal.pone.0253748 (PMC8375996; doi:10.1371/journal.pone.0253748)
Supplement: S1 Table — Note: Model includes the sugar content variable and the brand fixed effects as controls. SE: Standard error. + p < 0.10, * p < 0.05, ** p < 0.01, *** p < 0.001 Source: Authors’ own analyses and calculations based on data from Nielsen through its Mexico Consumer Panel Service (CPS) for the food and beverage categories for January 2012 –December 2015. The Nielsen Company, 2016. Nielsen is not responsible for and had no role in preparing the results reported herein. (DOCX) [file pone.0253748.s003.docx]

**S1 Table. First stage estimates for the price endogeneity**

| **Instrument** | **Coefficient**  **(SE)** |
| --- | --- |
| Sugar price index | 0.001 |
|  | (0.003) |
| Sugar price index*Producer #2 | -0.003 |
|  | (0.004) |
| Sugar price index*Producer #3 | -0.012*** |
|  | (0.003) |
| Sugar price index*Producer #4 | 0.113*** |
|  | (0.003) |
| Sugar price index*Producer #5 | 0.045*** |
|  | (0.005) |
| Sugar price index*Producer #6 | -0.003 |
|  | (0.003) |
| Sugar price index*Producer #7 | 0.002 |
|  | (0.003) |
| Tax implementation | 0.758 *** |
|  | (0.026) |
| F-statistic | 1203.8 |
| **Observations** | **1392** |
| Note: Model includes the sugar content variable and the brand fixed effects as controls. SE: Standard error. ^+^ p < 0.10, * p < 0.05, ** p < 0.01, *** p < 0.001 Source: Authors’ own analyses and calculations based on data from Nielsen through its Mexico Consumer Panel Service (CPS) for the food and beverage categories for January 2012 – December 2015. The Nielsen Company, 2016. Nielsen is not responsible for and had no role in preparing the results reported herein. | |
