# Supplementary material for: Simulating international tax designs on sugar-sweetened beverages in Mexico
Source: PLoS One. 2021 Aug 19;16(8):e0253748. doi: 10.1371/journal.pone.0253748 (PMC8375996; doi:10.1371/journal.pone.0253748)
Supplement: S2 Table — Note: Prices are calculated as quantity-weighted average prices. MP: Mexican pesos. Source: Authors’ own analyses and calculations based on data from Nielsen through its Mexico Consumer Panel Service (CPS) for the food and beverage categories for January 2012 –December 2015. The Nielsen Company, 2016. Nielsen is not responsible for and had no role in preparing the results reported herein. (DOCX) [file pone.0253748.s004.docx]

**S2 Table. Model fit for the volumetric one-Mexican Peso SSB tax in 2014 and 2015**

|  | **Taxed beverages** | | | | |  | **Untaxed beverages** | | | | |
| --- | --- | --- | --- | --- | --- | --- | --- | --- | --- | --- | --- |
|  | **Observed** | |  | **Simulated** | |  | **Observed** | |  | **Simulated** | |
| **Period** | **Prices** | **Mkt. Shares** |  | **Prices** | **Mkt. Shares** |  | **Prices** | **Mkt. Shares** |  | **Prices** | **Mkt. Shares** |
| Jan-14 | 9.104 | 19.255 |  | 9.104 | 19.255 |  | 2.034 | 19.012 |  | 2.034 | 19.012 |
| Feb-14 | 9.382 | 18.063 |  | 9.382 | 18.063 |  | 2.002 | 19.088 |  | 2.002 | 19.088 |
| Mar-14 | 9.325 | 17.777 |  | 9.325 | 17.777 |  | 2.054 | 19.015 |  | 2.054 | 19.015 |
| Apr-14 | 9.394 | 18.023 |  | 9.394 | 18.023 |  | 2.067 | 19.115 |  | 2.067 | 19.114 |
| May-14 | 9.465 | 18.200 |  | 9.465 | 18.200 |  | 2.099 | 18.782 |  | 2.099 | 18.782 |
| Jun-14 | 9.533 | 16.868 |  | 9.533 | 16.867 |  | 2.045 | 18.382 |  | 2.045 | 18.382 |
| Jul-14 | 9.446 | 17.385 |  | 9.446 | 17.385 |  | 2.018 | 17.950 |  | 2.018 | 17.949 |
| Aug-14 | 9.530 | 17.138 |  | 9.530 | 17.138 |  | 2.023 | 18.207 |  | 2.023 | 18.207 |
| Sep-14 | 9.404 | 17.576 |  | 9.404 | 17.576 |  | 1.999 | 18.084 |  | 1.999 | 18.084 |
| Oct-14 | 9.407 | 16.722 |  | 9.407 | 16.722 |  | 1.973 | 18.522 |  | 1.973 | 18.522 |
| Nov-14 | 9.252 | 17.354 |  | 9.252 | 17.354 |  | 2.012 | 17.974 |  | 2.012 | 17.974 |
| Dec-14 | 9.138 | 18.567 |  | 9.138 | 18.567 |  | 2.014 | 18.196 |  | 2.014 | 18.196 |
| Jan-15 | 9.194 | 18.894 |  | 9.193 | 18.894 |  | 2.015 | 19.725 |  | 2.015 | 19.725 |
| Feb-15 | 9.297 | 20.016 |  | 9.297 | 20.016 |  | 2.031 | 20.322 |  | 2.031 | 20.322 |
| Mar-15 | 9.337 | 17.612 |  | 9.337 | 17.612 |  | 2.039 | 18.525 |  | 2.039 | 18.525 |
| Apr-15 | 9.326 | 17.488 |  | 9.327 | 17.487 |  | 2.026 | 18.947 |  | 2.026 | 18.948 |
| May-15 | 9.459 | 17.224 |  | 9.459 | 17.224 |  | 2.106 | 18.815 |  | 2.106 | 18.815 |
| Jun-15 | 9.516 | 16.264 |  | 9.516 | 16.263 |  | 2.073 | 17.669 |  | 2.073 | 17.669 |
| Jul-15 | 9.561 | 16.671 |  | 9.561 | 16.671 |  | 2.030 | 17.751 |  | 2.030 | 17.751 |
| Aug-15 | 9.577 | 16.647 |  | 9.577 | 16.647 |  | 2.131 | 17.262 |  | 2.131 | 17.262 |
| Sep-15 | 9.529 | 17.093 |  | 9.529 | 17.093 |  | 2.169 | 16.519 |  | 2.169 | 16.518 |
| Oct-15 | 9.558 | 16.103 |  | 9.558 | 16.103 |  | 2.088 | 16.755 |  | 2.088 | 16.755 |
| Nov-15 | 9.484 | 17.654 |  | 9.484 | 17.654 |  | 2.089 | 16.814 |  | 2.089 | 16.814 |
| Dec-15 | 9.351 | 18.488 |  | 9.351 | 18.488 |  | 2.134 | 16.514 |  | 2.134 | 16.514 |
| Note: Prices are calculated as quantity-weighted average prices. MP: Mexican pesos. Source: Authors’ own analyses and calculations based on data from Nielsen through its Mexico Consumer Panel Service (CPS) for the food and beverage categories for January 2012 – December 2015. The Nielsen Company, 2016. Nielsen is not responsible for and had no role in preparing the results reported herein. | | | | | | | | | | | |
