# Supplementary material for: Simulating international tax designs on sugar-sweetened beverages in Mexico
Source: PLoS One. 2021 Aug 19;16(8):e0253748. doi: 10.1371/journal.pone.0253748 (PMC8375996; doi:10.1371/journal.pone.0253748)
Supplement: S3 Table — Note: Standard error in parentheses. Model includes brand fixed effects. Kid stands out for the presence of household members aged <13 years. + p< 0.10, * p < 0:05, ** p < 0:01, *** p < 0:001. Source: Authors’ own analyses and calculations based on data from Nielsen through its Mexico Consumer Panel Service (CPS) for the food and beverage categories for January 2012 –December 2015. The Nielsen Company, 2016. Nielsen is not responsible for and had no role in preparing the results reported herein. (DOCX) [file pone.0253748.s005.docx]

**S3 Table. Sensitivity analyses for the demand model**

|  | **Coefficient** |  | **Coefficient** |  | **Coefficient** |  | **Coefficient** |  | **Coefficient** |  | **Coefficient** |
| --- | --- | --- | --- | --- | --- | --- | --- | --- | --- | --- | --- |
| Mean utility |  |  |  |  |  |  |  |  |  |  |  |
| Sugar | 0.0185*** |  | 0.0186*** |  | 0.0185*** |  | 0.0185*** |  | 0.0186*** |  | 0.016**** |
|  | (0.000423) |  | (0.000441) |  | (0.000484) |  | (0.000424) |  | (0.000459) |  | (0.0003) |
| Price | -1.639*** |  | -1.642*** |  | -1.655* |  | -1.594*** |  | -1.706*** |  | -0.418*** |
|  | (0.356) |  | (0.291) |  | (0.669) |  | (0.320) |  | (0.415) |  | (0.006) |
| Price |  |  |  |  |  |  |  |  |  |  |  |
| Income | 0.156*** |  | 0.157*** |  | 0.158* |  | 0.151*** |  | 0.164*** |  | - |
|  | (0.0418) |  | (0.0344) |  | (0.0797) |  | (0.0371) |  | (0.0486) |  | - |
| Kid | -0.683** |  | -0.592*** |  | -0.793+ |  | -0.687* |  | -0.753* |  | - |
|  | (0.264) |  | (0.164) |  | (0.418) |  | (0.278) |  | (0.360) |  | - |
| Standard Deviation | 0.00569 |  | 0.000402 |  | 0.00777 |  | 0.000 |  | 0.0113 |  | - |
|  | (0.246) |  | (0.264) |  | (0.623) |  | (0.277) |  | (0.246) |  | - |
| Random-coefficients logit demand model | YES |  | YES |  | YES |  | YES |  | YES |  | NO |
| Draws for numerical integration | 1,000 |  | 2,000 |  | 5,000 |  | 1,000 |  | 1,000 |  | - |
| Fixed-effects seasonality | NO |  | NO |  | NO |  | YES |  | NO |  | NO |
| Additional instrument: prices from other cities | NO |  | NO |  | NO |  | NO |  | YES |  | NO |
| Observations | 1392 |  | 1392 |  | 1392 |  | 1392 |  | 1392 |  | 1392 |
| Note: Standard error in parentheses. Model includes brand fixed effects. Kid stands out for the presence of household members aged <13 years. + p< 0.10, * p < 0:05, ** p < 0:01, *** p < 0:001. Source: Authors’ own analyses and calculations based on data from Nielsen through its Mexico Consumer Panel Service (CPS) for the food and beverage categories for January 2012 – December 2015. The Nielsen Company, 2016. Nielsen is not responsible for and had no role in preparing the results reported herein. | | | | | | | | | | | |
