# Supplementary material for: Simulating international tax designs on sugar-sweetened beverages in Mexico
Source: PLoS One. 2021 Aug 19;16(8):e0253748. doi: 10.1371/journal.pone.0253748 (PMC8375996; doi:10.1371/journal.pone.0253748)
Supplement: S5 Table — Note: Prices are calculated as quantity-weighted average prices. MP: Mexican Pesos, PC-PD: Per capita Per Day. Source: Authors’ own analyses and calculations based on data from Nielsen through its Mexico Consumer Panel Service (CPS) for the food and beverage categories for January 2012 –December 2015. The Nielsen Company, 2016. Nielsen is not responsible for and had no role in preparing the results reported herein. (DOCX) [file pone.0253748.s007.docx]

**S5 Table. Tax effect in 2014 and 2015: Reformulation scenario of sweetness unchanged with null marginal cost adjustments**

|  |  |  | **Effect compared to No Tax in place** | | | | |
| --- | --- | --- | --- | --- | --- | --- | --- |
|  | **No tax in place** |  | **United-Kingdom tax design** | |  | **South-Africa tax design** | |
|  |  |  |  |  |  |  |  |
| **Outcomes** |  |  | **Lead firms** | **All firms** |  | **Lead firms** | **All firms** |
| Prices ($ MP) | 8.17 |  | 1.21 | 1.14 |  | 0.83 | 0.66 |
| Prices (%) |  |  | 14.77 | 13.93 |  | 10.16 | 8.04 |
| Tax Pass-through level (%) |  |  | - | - |  | - | - |
| PC-PD Volume (mL) | 204.14 |  | -37.75 | -35.95 |  | -25.41 | -21.14 |
| Volume (%) |  |  | -18.49 | -17.61 |  | -12.45 | -10.36 |
| Implied price elasticity of demand (volume) |  |  | - | - |  | - | - |
| PC-PD Sugar (grams) | 21.30 |  | -4.25 | -4.52 |  | -7.49 | -8.01 |
| Sugar (%) |  |  | -19.95 | -21.22 |  | -35.16 | -37.62 |
| Implied price elasticity of demand (sugar) |  |  | - | - |  | - | - |
| Tax revenue per capita per year ($ MP) |  |  | 58.91 | 56.85 |  | 37.94 | 34.11 |
| Note: Prices are calculated as quantity-weighted average prices. MP: Mexican Pesos, PC-PD: Per capita Per Day. Source: Authors’ own analyses and calculations based on data from Nielsen through its Mexico Consumer Panel Service (CPS) for the food and beverage categories for January 2012 – December 2015. The Nielsen Company, 2016. Nielsen is not responsible for and had no role in preparing the results reported herein. | | | | | | | |
