# Supplementary material for: Simulating international tax designs on sugar-sweetened beverages in Mexico
Source: PLoS One. 2021 Aug 19;16(8):e0253748. doi: 10.1371/journal.pone.0253748 (PMC8375996; doi:10.1371/journal.pone.0253748)
Supplement: S1 File — (DOCX) [file pone.0253748.s008.docx]

**S1 File**

**SSB tax in the United Kingdom and South Africa for the Mexican setting**

In the United Kingdom (UK) since 2018, SSB are subject to an excise tax according to sugar content thresholds. No levy tax applies to SSB with <5 sugar grams per 100 ml. Meanwhile, the tax levy is £0.18 per liter (low levy) for SSB with 5-8 sugar grams per 100 ml and £0.24 per liter (high levy) for SSB with >8 sugar grams per 100 ml [1]. According to Scarborough et al. [1], average pre-tax prices in the UK were £3.19 per liter of low-levy SSB and £2.51 per liter of high-levy SSB. By comparing these prices to the tax levies, the low tax levy and high tax levy are equivalent to 5.64% and 9.56% of their corresponding SSB pre-tax price, respectively. Based on these rates and SSB pre-tax prices from Nielsen CPS [2], we apply the UK tax design for the Mexican setting in two ways. In the first approach, we use the UK tax rates as is to translate to Mexican pesos (MP). In 2012 and 2013 in urban Mexico, the average pre-tax price was MP$7.99 per liter of SSB with 5-8 sugar grams per 100 ml (low levy) and MP$8.50 per liter of SSB with >8 sugar grams per 100 ml (high levy) [2]. Therefore, we set respective excise taxes of MP$0.45 and MP$0.81 per liter of low- and high-levy SSB in Mexico to be consistent with the ratio between pre-tax prices and the tax levy in the UK. This first approach only allows a comparison of the Mexico to UK tax designs based on their set tax rates. Therefore, this approach does not allow us to distinguish whether differences in results are due to different tax rates or due to tax design (volumetric vs. tiered sugar-density). Therefore, we applied a second approach that adapts the UK tax rates such that the resultant tax rate would match that of the Mexico volumetric tax (one MP per liter). Under this second approach, the tax is MP$0.755 and MP$1.01 per liter for the low- and high-levy SSB as in the UK tax design, respectively.

In South Africa (ZA), a tax of ZAR$0.021 per additional sugar gram beyond 4 sugar grams per 100 ml applies to SSB since April 2018 [3]. Stacey et al. [4] point out that this tax represents between 10 and 11% of the price of the top soft drink brand. We adapted the ZA tax design for the Mexican setting following an approach equivalent to the UK tax design above. The sugar content and pre-tax price for the top soft drink brand in urban Mexico are 106.8 grams and MP$9.18 per liter, respectively. Thus, for the first approach, to equalize the ZA tax design in Mexico, we used a sugary tax of MP$0.014 per sugar gram. However, it is worth noting that our analytical data for Mexico do not include SSB with <4 sugar grams per 100 (see Fig 2), which represents SSB exempted from the sugary tax in ZA. For the second approach where we adapt the ZA tax rate such that the resultant tax rate would match that of the Mexico volumetric tax (one MP per liter), this was set at MP$0.0156 per gram of sugar density beyond 4 grams as in the ZA tax design. Fig 1 presents the tax rates under all tax designs of interest in this study.

**Tax effect on purchases (volume and sugar) and tax revenue**

To estimate the effect for each SSB tax of interest on purchases in terms of volume in 2014-15, we use the simulated price information with and without the tax of interest along with equation (S.1):

|  | $\Delta Purchases=\sum_{t}^{T} Q_{t}\sum_{j=1}^{J} [S_{jt}\left( \tilde{p}_{t}^{\mathrm{NoTx}}, X; \theta\right)- S_{jt}\left( \tilde{p}_{t}^{\mathrm{Tx}}, X^{Tx}; \theta\right)];$ | (S.1) |
| --- | --- | --- |

where $\Delta Purchases$ is the overall change in purchases measured in purchase volume in 2014-15, $Q_{t}$ is the market size in terms of purchase volume in liters in month t in years 2014-15, and $S_{jt}\left( \tilde{p}_{t}^{\mathrm{NoTx}}, X; \theta\right)$ and $S_{jt}\left( \tilde{p}_{t}^{\mathrm{Tx}}, X^{Tx}; \theta\right)$ are the estimated market shares for beverage $j$ in the absence or presence of the SSB tax of interest, respectively. The matrix of products’ characteristics $X^{Tx}$ includes the sugar information under the different reformulation scenarios for each tax design of interest. When we assume no reformulation, we retain the original variable of sugar that we used to run the demand model. Under the reformulation scenario of sweetness unchanged, we keep using the original variable of sugar, assuming that consumers do not experience any utility change linked to the substitution of sugar for artificial non-caloric sweeteners. Under the reformulation scenario of sweetness reduction, we adjusted the sugar information downwards in $X^{Tx}$ according to the assumed sugar reduction for the UK or ZA sugar-density taxes.

We estimated the sugar reduction from SSB under each tax design of interest using equation (S.2):

|  | $\Delta Sugar=\sum_{t}^{T} Q_{t}\sum_{j=1}^{J} [{Sugar}_{j}*S_{jt}\left( \tilde{p}_{t}^{\mathrm{NoTx}}, X; \theta\right)- {{Sugar}_{j}^{Tx}*S}_{jt}\left( \tilde{p}_{t}^{\mathrm{Tx}}, X^{Tx}; \theta\right)];$ | (S.2) |
| --- | --- | --- |

where ${Sugar}_{j}$ and ${Sugar}_{j}^{Tx}$ represent the original sugar variable in equation (1) and the sugar variable under the reformulation scenarios, respectively. Within each of the tax designs in the UK or ZA, ${Sugar}_{j}^{Tx}$ will be the same under any reformulation scenario due to equation (S.2) allows us to estimate the sugar reduction resulting from the tax effect through prices and reformulation. The remaining variables in equation (S.2) preserve the same interpretation as in equation (S.1).

To estimate the tax revenue under each tax design, we use equation (S.3):

|  | $Tax Revemue=\sum_{t}^{T} Q_{t}\sum_{j=1}^{J} [{Tx}_{jt}^{Ex,c}*S_{jt}\left( \tilde{p}_{t}^{\mathrm{Tx}}, X^{Tx}; \theta\right)];$ | (S.3) |
| --- | --- | --- |

where ${Tx}_{jt}^{Ex,c}$ represents the excise tax for taxed beverage $j$ according to the tax design in country $c$. ${Tx}_{jt}^{Ex,c}$ for taxed beverages will equal one for the case of the volumetric one-MP tax. For tax designs as in the UK or ZA, ${Tx}_{jt}^{Ex,c}$ is a function of the beverage $j$’s sugar content. Under each of our assumptions linked to the reformulation scenarios, we adjust ${Tx}_{jt}^{Ex,c}$under tax designs in the UK or ZA according to ${Sugar}_{j}^{Tx}$, as introduced in equation (S.2).

**References**

1. Scarborough P, Adhikari V, Harrington RA, Elhussein A, Briggs A, Rayner M, et al. Impact of the announcement and implementation of the UK Soft Drinks Industry Levy on sugar content, price, product size and number of available soft drinks in the UK, 2015-19: A controlled interrupted time series analysis. Popkin BM, editor. PLOS Med. 2020;17: e1003025. doi:10.1371/journal.pmed.1003025

2. The Nielsen Company. Mexico Consumer Panel Service (CPS) for the food and beverage categories for January 2012 – December 2015. 2016.

3. Global Food Research Program UNC. Sugary drink taxes around the world. 2019.

4. Stacey N, Mudara C, Ng SW, van Walbeek C, Hofman K, Edoka I. Sugar-based beverage taxes and beverage prices: Evidence from South Africa’s Health Promotion Levy. Soc Sci Med. 2019. doi:10.1016/j.socscimed.2019.112465
