# Supplementary material for: Simulating international tax designs on sugar-sweetened beverages in Mexico
Source: PLoS One. 2021 Aug 19;16(8):e0253748. doi: 10.1371/journal.pone.0253748 (PMC8375996; doi:10.1371/journal.pone.0253748)
Supplement: Attachment — Submitted filename: Response to Reviewers.docx [file pone.0253748.s009.docx]

Reviewers' comments:

**Reviewer #1:** 

This is an important article that examines alternative sugar-sweetened beverages tax scenarios in Mexico. The authors suggest that a sugar-density tax, which encourages product reformulation, would better tackle obesity in Mexico by producing a larger effect in demand than the volumetric tax. The results from this study are relevant throughout the Latin American region and elsewhere, as many countries are implementing strategies to reduce the consumption of these beverages. I do not have major comments regarding the methodology. However, I have suggestions about topics that should be added to the discussion section. In addition, I have a few minor comments throughout the manuscript.

*Response: Thank you for your helpful suggestions and comments to improve this manuscript. We respond to each in turn. In the new version of the manuscript, you will find the respective changes derived from your comments and some other updates to address comments by the second reviewer. Due to adjustments in the demand model specification, we updated the results in tables and figures other than Table 1 and Figures 1 and 2. In addition to all changes as responses to the reviewers’ comments, we fixed some inconsistencies that we identified while re-reading our manuscript. First, we re-wrote equation (1) by removing the term* $\varepsilon_{ijt}$ *when this equation enters equations (2) and (3), and thus to be consistent with the logit formula for these two equations. This correction is related to notation rather than the empirical application of our demand model. Second, we specified that the tax amount that enters equation (6) is in terms of per liter, which is consistent with our explanation when we introduced that tax as an element of the first-order conditions. Third, for the reformulation scenarios in ZA, we clarified that marginal cost changes are based on the tax burden difference by comparing this burden with and without reformulation. This approach is equivalent to our explanation for the UK tax design and what we have been simulating. In general, our overall findings and conclusions remained similar to our first submission.*

1. The analysis is focused on Mexican urban household purchases, considering the 20-liter jars of plain drinking water as an “outside option”. The market share for this type of product has been found to be different among urban versus rural households. I think this should be acknowledged in the discussion section as this could have implications in the interpretation of the models.

*Response: To include the relevance of the urban-rural difference and its potential implications, we updated our study’s limitations as follows:*

*“Thus, we cannot generalize our findings due to existing differences between rural and urban settings. In terms of market composition, a lower proportion of rural households directly reports bottled water as their main drinking water source [23]. Moreover, there was a lower magnitude of both SSB price increases and SSB purchases drops after the tax implementation in the rural areas compared to urban areas [11,48,49]. In light of these differences, we might expect a lower tax effect if we were able to implement our model in rural areas, but without empirical data, this is difficult to know with certainty.”*

2. Authors acknowledge that the current tax in Mexico is unlikely to change. Meanwhile Mexico has recently implemented a front-of-packaging labeling policy which is likely to encourage beverage reformulation. It think the authors could include a few sentences in the discussion about the possible synergy between these two strategies on demand and consumption of SSBs.

*Response: We thank the reviewer for this suggestion in light of the public health relevance of the front-of-packaging labeling policy. Hence, we included the next sentence as part of the last paragraph in the section of “Discussion”:*

*“Moreover, the SSB tax re-design toward sugar-density taxes might reinforce the potential reformulation and subsequent sugar intake reduction linked to the front-of-packaging labeling policy for food and beverages in effect in Mexico since October 2020[55].”*

3. I think it would be worth to include a sentence of how the authors defined SSBs and non-SSBs for the purpose of this study. Were they defined according to a grams/100 mL threshold? Added sugar content? Any sugar content?

*Response: In the sections of “INTRODUCTION” and “Data”, we updated the next sentences to provide a better explanation of which products compose each beverage category.*

*In the “INTRODUCTION”, we specified that the volumetric tax applies to any SSB with any positive value of added sugar:*

*Previously, the sentence was:*

*“To help temper the high SSB consumption and curb the rise in obesity and diabetes in Mexico, the government implemented a specific volumetric excise tax of one Mexican peso (MP) per liter on SSBs starting in January 2014.”*

*The new version of the sentence is:*

*“To help temper the high SSB consumption and curb the rise in obesity and diabetes in Mexico, the government implemented since January 2014 a specific volumetric excise tax of one Mexican peso (MP) per liter of SSBs with any positive added sugar content* *(herein “volumetric one-MP tax”)”*

*In the section of “Data”, we expanded our explanation regard the beverages that compose the untaxed and taxed categories*

*Previously, the sentence was:*

*“We focused on the non-dairy and non-alcoholic beverage market composed of plain water and diet SSB (representing untaxed beverages), and SSBs (subject to the tax).*

*The new version of the sentence is:*

*“We focused on the non-dairy and non-alcoholic beverage market composed of plain water and diet SSB, which by design do not contain sugar and represent the untaxed beverages, and SSBs with any level of added sugar and thus subject to the tax.”*

*Regarding our econometric approach, the sugar information enters the model in terms of grams per liter as we defined it right after equation (1).*

4. Page 6, Line 111: It would be worth to include a line saying that this panel captures the decline in sugar content on beverages after 2014 tax implementation

*Response: We thank the reviewer for this comment, but according to our best knowledge, there is no evidence to date showing sugar content reduction linked to the implementation of the 2014 SSB volumetric tax in Mexico. Reformulation is unlikely under this tax design since SSBs are subject to the same tax amount regardless of their added sugar content. However, we highlight that the panel data for the full period provide market data before and after the tax implementation. We aimed at extending these arguments by updating the next sentences:*

*Regarding exploiting pre- and post-tax data, we extended the first sentence in the section of “Data” when we introduce the Nielsen CPS data. Thus, the updated version of this sentence is*

*“The main source of information for our analysis is the Nielsen Mexico Consumer Panel Service (Nielsen CPS) [22] from 2012 to 2015 so that we have access to data for both pre-tax periods (i.e., 2012-2013) and post-tax periods (i.e., 2014-2015).”*

*Regarding the topic of reformulation under the volumetric SSB tax in Mexico, we included the next sentence in the paragraph focused on the potential effects linked to sugar-density taxes in the section of “INTRODUCTION”:*

*“**Conversely, the current volumetric one-MP SSB tax in Mexico is unlikely to lead to reformulation since the tax burden is independent of the sugar content across any SSB with added sugar”*

5. Page 9, line 175: I suggest that the authors review the manuscript for consistency on how the one-Mexican Peso volumetric tax is referred to throughout the manuscript and tables

*Response: To guarantee consistency, we add the next specification when we, first, introduce the one-Mexican Peso volumetric tax in the second paragraph of the “INTRODUCTION”:*

*“(herein “volumetric one-MP tax”)”*

*We reviewed the manuscript and updated any mention to the current SSB tax as “volumetric one-MP tax”*

6. Page 11, line 212: I suggest that authors only use abbreviations if the terms will be mentioned more than 3 times throughout the manuscript to improve readability

*Response: We have done as the review suggests. For example, we no longer use the abbreviation FOC and use instead the full definition.*

7. Page 15, Figure 2: I recommend that the authors re-label the figure to indicate that the Y-axis refers to Percentage of beverage

*Response: We re-labeled the Y-axis accordingly.*

8. Page 17, Table 2: Please use a more clear label to refer to the “Kid” coefficient in the table. Alternatively, the authors could explain this label on a footnote

*Response: In the footnote in Table 2 and supplementary tables for the sensitivity analyses for the demand model specification, we included the next sentence as the footnote “a”:*

*^a^“Kid” stands out for the presence of household members aged <13 years”*

9. Page 18, line 326: Results suggest a negative marginal cost for plain water and authors suggest that it is possible that producers might be obtaining a subsidy or credit to produce plain water. I suggest that they elaborate on this and provide a reference

*Response: After our explanation of a potential subsidy or credit for plain water production, we include the next sentence:*

*“For example, one of the leading companies in the non-dairy and non-alcoholic beverage market pays USD $0.10 for 1,000 liters of water to the Mexican federal government [44]; meanwhile, Mexican households pay about USD $0.70 for the same amount of water [45].”*

**Reviewer #2**

*Response: Thank you for your helpful suggestions and comments to improve this manuscript. We respond to each in turn. In the new version of the manuscript, you will find the respective changes derived from your comments and some other updates to address comments by the second reviewer. Due to adjustments in the demand model specification, we updated the results in tables and figures other than Table 1 and Figures 1 and 2. In addition to all changes as responses to the reviewers’ comments, we fixed some inconsistencies that we identified while re-reading our manuscript. First, we re-wrote equation (1) by removing the term* $\varepsilon_{ijt}$ *when this equation enters equations (2) and (3), and thus to be consistent with the logit formula for these two equations. This correction is related to notation rather than the empirical application of our demand model. Second, we specified that the tax amount that enters equation (6) is in terms of per liter, which is consistent with our explanation when we introduced that tax as an element of the first-order conditions. Third, for the reformulation scenarios in ZA, we clarified that marginal cost changes are based on the tax burden difference by comparing this burden with and without reformulation. This approach is equivalent to our explanation for the UK tax design and what we have been simulating. In general, our overall findings and conclusions remained similar to our first submission.*

Referee report on
“Simulating international tax design on sugar-sweetened beverages in Mexico”,
By Juan Carlos Salgado & Shu Wen Ng.

This research article aims at simulating the impact of various tax schemes on the Mexican market for non-dairy and non-alcoholic beverages. The authors leverage household scanner data to calibrate a structural econometric model of supply and demand. The scanner data spans the period 2012-2015 that includes the implementation of one Mexican Peso (MP) tax on all beverages containing less than 1g of sugar per L. They first use the estimated model to simulate the counterfactual market equilibrium that would prevail if Mexico had implemented the recent UK and South-African tax schemes that make the tax rate function of the sugar content of beverages (with a progressive linear scheme for South-Africa and a discrete scheme distinguishing low- and high-sugar products for the UK). They find little difference with the estimated impacts of the one-MP tax: the reductions in quantities and sugar intakes are slightly lower (around -15% vs. -18% for the one-MP tax). They then explore the consequences of reformulation strategies that producers would be likely to implement if sugar content were taxed. Two strategies are simulated (1) a substitution of sugar ingredients with artificial sweeteners that would leave unchanged the taste of products (2) a mere reduction in sugar content. They find much larger reduction in sugar intakes under these scenarios (about -40% to -45%).

Overall, I find that the research question is interesting and important for the public health community. Soft-drink taxes have now been adopted in many jurisdictions, with different tax designs, market structures and consumer preferences. Hence, any comparative approach can help identify the best tax schemes in terms of public health, consumer welfare and distributional effects. This study has therefore the potential to contribute to the literature. Its main strength is that the authors implements up-to-date techniques to model the beverage market.
However, I have important concerns and suggestions regarding the internal validity of the empirical analysis, the scenarios that are simulated and the discussion of the results.

Empirical model
The authors adopt an Empirical Industrial Organisation (EIO) approach that has been used inter alia in studies of soft-drink taxes (Bonnet and Réquillart 2013) and nutritional labeling (Allais, Etilé et al. 2015). In simplified terms, to model a market, one must identify a demand curve and a supply curve. A key issue is that the supply curve is difficult to identify directly because data on firms’ production technologies, marginal production costs and margins are often unavailable. Berry, Levinsohn et al. (1995) proposed to overturn this problem through a two-step approach. Demand functions for the various products are estimated first, using available market data (prices and market shares). Then supply functions are calibrated by assuming that the market is in equilibrium and has a specific structure in terms of competition. Here, the authors assume a Bertrand-Nash oligopolistic competition between a limited set of producers (as in Allais et al., 2015), and they do not model the vertical relationships between retailers and producers (unlike, e.g., Bonnet & Réquillart, 2013). The main target parameters for the supply functions are the unit costs of production, which are identified by inverting the first-order conditions of profit maximisation by the producers. The authors implement this approach using a random-coefficient logit (RCL) model for modelling consumer demand (available in a Stata package, see Vincent, 2015).
The main threat to the internal validity of this approach is that the demand functions may be mis-specified and are difficult to identify. I now comment more precisely on these points:

1. The demand model assumes that the relevant market for Sugar-Sweetened Beverages (SSB) includes non-alcoholic non-dairy beverages and takes 20-liter jars of plain drinking water as the outside option:
   a. The products are essentially differentiated in terms of brand (product fixed-effect in equation (1)) and sugar-content. However, the sugar-content appears to be product-specific and invariant over time, so that its marginal utility cannot be identified separately from the product fixed-effect. The only way to achieve identification is to have a brand fixed-effect (e.g. Coca-Cola) and introduce additional product attributes such as the sugar content (e.g. Light/Zero vs. regular) or the packaging, see Dubois, Griffith et al. (2020).

*Response: For our analytical data, we have regular and light products for some brands that allowed us to identify the coefficient on sugar. We acknowledge we did not provide a proper explanation of this variation. Thus, we extended our description of the relevant fixed effects in equation (1) as follows:*

*“**For the sake of simplicity in notation, we specify* $\xi_{j}$ *at the product level, however, these fixed effects enter the model as brand fixed-effects. Eight out of the 29 products in the inside option correspond to brands with light and regular versions that allowed us to identify the coefficient on sugar.”*

b. However, the sugar-content may have varied over time, following actual product reformulations by producers. The paragraph (P6 L109-112) discussing the nutritional information obtained at the product level should be more precise about this: what is the “average sugar content at the product level”? (P6, L111)

*Response: We acknowledge our description regarding nutritional information is not precise enough. This information is time-invariant at the product level and comes from the UNC Mexican Nutrition Fact Panel, which collects the most recent available information from different datasets spanning different time periods. . Thus, the average does not correspond to variations over time. Instead, this average comes from those products with variations in flavors, such as flavored waters and juices. It is possible that some reformulation occurred, and the present nutritional data does not reflect it. However, no peer-reviewed studies to date have documented reformulations across the beverage market linked to the SSB tax in Mexico. Arguably, reformulation due to Mexicos’ SSB volumetric tax is low since SSBs are subject to the same tax amount regardless of their added sugar content. We aimed at extending these arguments by updating the relevant sentences.*

*Regarding the average sugar, we updated the last sentences for the paragraph where we introduced the nutritional information in the section of “Data”. Thus, the updated version of this sentence is:*

*“We complemented purchase and price information from Nielsen CPS with information on time-invariant sugar content at the SSB brand level from the UNC Mexican Nutrition Fact Panel[28].* *This panel collects the most recent available information from products’ pictures in different datasets and product-specialized websites (e.g., Redcap) spanning different time periods, as explained elsewhere [10]. We used sugar content at the product level as an SSB’s attribute of interest for our demand model. For products in our analytical data with variations in flavors such as flavored waters and juices, we used their average sugar content.”*

*Regarding the topic of reformulation under the volumetric SSB tax in Mexico, we included the next sentence in the paragraph focused on the potential effects linked to sugar-density taxes in the section of “INTRODUCTION”:*

*“Conversely, the current volumetric SSB tax in Mexico is unlikely to lead to reformulation since the tax burden is independent of the sugar content across any SSB with added sugar.”*

c. Do we have any information that may help to justify the exclusion of non-dairy products from the relevant market for SSB? Do urban households have access to tap water?

*Response: We interpreted the reviewer's previous question as the need to justify the exclusion of dairy products rather than non-dairy products. We excluded the former products because Nielsen CPS did not include dairy information up to October 2012. Thus, we did not have complete dairy information for the full analytical period in our study. To clarify this, we included the next sentence when we introduce the Nielsen CPS data in the section of “Data”:*

*“We excluded dairy products because of missing information from January 2012 to September 2012.”*

*In Mexico, tap water might be perceived as an unhealthy/unsafe option (e.g., unpurified water) and thus is not a common option across households. According to the national survey of health and nutrition in 2018, about 10% of urban households directly report tap water as their main drinking water source, which was less than half the proportion compared to rural households. At the national level, the proportion of households mainly relying on tap water reaches 20%. Thus, we include the next sentence in our description of the relevant market in the section of “Data”:*

*“Moreover, we did not focus on tap water because this option is not dominant in urban Mexico due to concerns/mistrust around potability, with only about 10% of households directly reporting tap water as their main drinking water source [23] (this proportion can reach 20% at the national level[24]).”*

d. I understand that the price of the outside option is set at 0 (P7 L138). Were the product prices specified in terms of difference with the price of the outside option?

*Response: We were unaware of the adjustment of inside options’ prices with respect to the outside option’s price. For the new version of the demand model specification, inside options’ prices enter the model as the difference with the outside option’s price. We specified the price adjustment with the next sentence in the section of “Demand model”:*

*“Price information in vector p is in terms of the difference of the price of the outside option in market* $t$*”.*

2. I understand that a market is one month: the identification does not attempt to exploit cross-sectional variations, e.g variations in market structure between Mexican cities. In addition, the model is estimated using the data aggregated at a monthly level, not the individual-level data.
   a. Would it be possible to estimate directly mixed logit models of household choices (without aggregation)? Or it does not make sense because the survey is constructed to be exploited at an aggregate level only?

*Response: Because we are interested in analyzing several time periods (i.e., 48 months), we followed the approach consistent with other logit demand models where authors aggregate household data at the market level to analyze data from several years. Hence, we included the next sentence when describing the Nielsen CPS data:*

*“The aggregation of household data at the market level is a common approach in other studies on logit demand models analyzing several years of data (e.g., see Bonnet and Requillart[25], Liu, Lopez, and Zhu[26], and Zhu, Lopez, and Liu [27]). We are interested in analyzing the full data from 2012 to 2015 because we exploit the SSB price change after the tax implementation in January 2014* *(see S1 Fig in the supplemental appendix S1 File) as the main source of variation to identify the coefficients on prices.”*

b. The estimated standard-deviation of random price coefficient is close to zero and not significant. This is not surprising because the identification of the random-coefficient distribution in RCL relies essentially on price variations that must be large or rather uncorrelated between products (see for instance assumption (12) in Fox, il Kim et al. (2012)). Here, the two main source of identifying variations are the one-MP tax and the time variations in sugar costs. Hence, the price variations that are relevant for identification are likely to be correlated across products. In addition, the within-product price variations are not large.

*Response: For sensitivity analyses, we used prices from other cities as instruments (as explained in detail below) that are supposed to introduce an additional source of variation across both taxed and untaxed products. The inclusion of these prices as part of the set of instruments increased the point estimate of the standard deviation for prices (see S3 Table in the supplemental appendix S1 File); however, it remained statistically insignificant. Thus, we consider that these findings indicate that households’ observed characteristics in our model (i.e., income and presence of kids) drive price preferences. We included this description when we described estimates in Table 2.*

c. It would therefore be useful to complete this analysis by the estimation of standard logit model, to check the stability of the estimated price and sugar coefficients. This standard logit model can be estimated by regressing the log market shares (log(s_jt)-log(s_0t)) on the covariates, controlling for product fixed-effects. Note that this shows that identification of the taste for sugar requires that sugar-content display variations within products. Otherwise, it drops when one controls for the product fixed effects.

*Response: We included the estimates of the standard logit model accounting for the price endogeneity in S3 Table in the supplemental appendix S1 File 1. By comparing estimates from this model to the BLP demand model, we found a larger estimate (in absolute value) for the price coefficient for the latter model while the coefficient on sugar was comparable across models. As specified above, we were able to identify the sugar coefficient due to variations within brands. We hypothesize that the larger price coefficient in our random-coefficient logit demand model corresponds to differences in responses to price changes across households that vary in demographics. Meanwhile, the standard logit model is not able to account for these differences. Moreover, our findings go in the same direction as estimates from another study by Nevo.^[1]^ Thus, we included the next explanation at the bottom of the section of “Demand model estimates and model fit” when describing findings from the standard logit demand model:*

*“Moreover, we ran a standard logit demand model accounting for the price endogeneity and no random coefficients for comparability reasons. We found that coefficients on prices and sugar from the* *random-coefficients demand model were, respectively, larger (in absolute terms) and similar compared to their counterparts in the standard logit demand model. This is consistent with findings by Nevo [42], who found larger estimated coefficients on prices when using random coefficients and comparable estimates for products’ attributes with no random coefficients in either model (i.e., random-coefficients demand logit or standard demand logit). The difference in estimates across models in our study might arise from the random coefficients capturing larger dis-utility attached to price increases across households with different characteristics, which by design is overlooked in the standard logit model.”*

d. One way to have more variability is to leverage variations between cities. As local markets have different structures, there is a variability that is helpful for instrumenting the prices and for identification (see Etilé, Lecocq et al. (2020) for evidence on market heterogeneity in soft-drink tax incidence).

*Response: We adopted this approach by retrieving monthly price data from the National Institute of Statistics and Geography (termed as INEGI in Spanish), which uses these data to calculate the urban consumer price index. INEGI provides price information at the product level and specifies the city from which this information was collected. Thus, we used price information from cities in the data by INEGI that are not part of Nielsen CPS and matched this price information at the product level. In S3 Table for the sensitivity analyses in the supplemental appendix (S1 File), we showed that estimates of the demand model were robust to the inclusion of prices from other cities as part of the set of instrumental variables. We did not use this set as our preferred set of instrumental variables because most of the price-related coefficients were not statistically insignificant when using 5,000 draws of households’ characteristics for the demand model. However, point estimates of the demand model and simulations remained similar to our preferred model specification.*

e. More powerful instruments may perhaps be obtained by interacting the sugar content of products at baseline (in 2012) with the time variations in the producer price index for sugar.

*Response: As specified above, we do not have variation in sugar content over time. Thus, we preferred to continue using the interactions of producer price index sugar with the producers’ dummies. Moreover, we used prices from other cities (as explained above), which impacts tax and untaxed beverages, as part of the set of instrumental variables and we found that our results were robust.*

3. A key identifying assumption is that the one-MP tax did not affect consumer tastes. Hence, the tax increase can be used as a strong instrument for prices.
   a. This is a debatable assumption that should be discussed in the presentation of the demand model (P7) and in the discussion of the identification strategy (P8). A soft-drink tax may impact consumer tastes by making the health risks of SSB consumption more salient and, over the long-term, by denormalising soft-drinks. These non-price effects are likely to be observed primarily in the upper classes (see the example of Tobacco)

*Response:*

*We acknowledge our instrument related to the tax implementation lacks a proper explanation regarding its validity. We agree with the reviewer that taxes with a public-health foundation can impact purchase decisions through changes in health awareness and that these changes are more likely across the upper classes. This might be the case for the SSB tax in Chile, where larger SSB purchase drops took place across more affluent households. Conversely, empirical evidence shows an inverse pattern in Mexico, with low socio-economic households reporting the largest drop in SSB purchases, which was consistent with previous findings of larger price-sensitive demands for SSB for these households. Non-price effects linked to the SSB tax in Mexico are possible but unlikely to be stronger than the price effects. Hence, we included the next description when discussing the validity of the tax implementation as an instrument:*

*“**A threat to the validity of this instrument is that the SSB tax might lead to an awareness change in the negative health effects linked to SSB, and thus a non-price effect might drive the SSB purchase reductions. Teng et al. [39] suggested this could be the case for the SSB tax in Chile, where SSB reductions were larger among well-off households.* *We cannot properly ascertain if there is indeed a signaling effect and to what degree, but the potential signaling effect is likely to play a smaller role compared to the price effects in Mexico since previous quasi-experimental assessments show that, after the SSB tax implementation, poor households decreased their SSB purchases (both in absolute and relative terms) to a larger extent compared to well-off households[9]. These findings are consistent with previous evidence of larger price-sensitive demands for SSB among more socio-economic-deprived households in Mexico[40].”*

*Further, in the limitations of our study, we discussed the potential direction of the bias in the presence of a signaling effect linked to the SSB tax and analyzed the existing evidence on this topic. Hence, we aggregated the next sentences as part of our study’s limitations:*

*“**Second, in the presence of a signaling effect linked to the SSB tax, we would overestimate the tax effect attributable to price increases. A cross-sectional study based on 2016 data [50] showed that awareness of the SSB tax was more predominant across people from high-income and urban households. Greater awareness of the tax was associated with a higher probability of self-reporting a reduction in SSB consumption. However, the authors stated that this association does not entail causality due to potential unobservable variables linked to the SSB tax awareness[50]. Surprisingly, a larger proportion of people from high-income households aware of the SSB tax believed that the tax was not effective in reducing SSB consumption [50]. In light of these findings and previous evidence showing a negative relationship between SSB purchase reductions and households’ socio-economic status [9], consistent with demand price elasticities across households [40], we consider that price increases drove the reduction in SSB purchases.”*

b. An alternative strategy is to use the pre-tax data to calibrate the model, and then compare its predictions under the one-MP, the UK and the South-African scenarios. The authors should test the robustness of their main results to a switch to this alternative strategy. This would also be a means of conducting a proper test of model fit: does the model predict accurately what happened after the implementation of the one-MP tax?

*Response: We thank the reviewer for this suggestion. However, we did not pursue it for methodological reasons. First, when we ran the demand model using only pre-tax data, we found that point estimates were similar to our findings using the full data; however, while the coefficient on sugar remained statistically significant, that was not the case for coefficients on prices, which might be the result of lack of variability in the price data limited to only the pre-tax period combined with the smaller sample size. Second, and related to the previous comment/response for comment 2a above, we included S1 Fig in the supplemental appendix (S1 File) focused on SSB prices over time. This graph shows that the most important price variation took place in January 2014 (i.e., the tax implementation). We think this source of variation allowed us to identify the price coefficient.*

4. Miscellaneous points:
   a. The presentation of the inference method (P8 L147-152) lacks clarity. The important element in the approximation (3) is that the household demographics and unobservable characteristics are drawn simultaneously. From my own experience, the number of draws – here set to 500 – can have an impact on the estimated parameters. It is therefore necessary to provide evidence that increasing the number of draws to 1,000, 2,000, 5,000 does not improve the precision of the estimates.

*Response: We thank the reviewer for the proper explanation of the draws in equation (3). Thus, we re-wrote part of our description of equations (2) when we introduce the distributions* $P_{D}\left( D \right)$ *and* $P_{v}\left( v \right)$ *as follows:*

*“Households’ observed and unobserved characteristics are drawn simultaneously from the respective distributions* $P_{D}\left( D \right)$ *and* $P_{v}\left( v \right)$*.”*

*Previously, this sentence was:*

*“The distribution of households’ observed and unobserved characteristics is* $P_{D}\left( D \right)$ *and* $P_{v}\left( v \right)$*”*

*Regarding the number of draws for equation (3), we updated the relevant sentences as follows:*

*“We set an N equal to 1,000 that is the number of draws of* $v_{i}$ *from* $P_{v}\left( v \right)$ *and* $D_{i}$ *from* $P_{D}\left( D \right).$ *In practice for* $D_{i}$*, we randomly drew three sets of 1,000 urban households from each quarterly round of the National Survey of Occupation and Employment.”*

*As specified above, our baseline model specification is based on 1,000 draws rather than 500 draws as in our manuscript's first submission. We made this change due to one of the price-related coefficients (other than the standard deviation) was not statistically significant when using 500 draws. In contrast, the demand model was robust to 1,000, 2,000, and 5,000 draws, as shown in S3 Table in the supplemental appendix (S1 File) focused on the sensitivity analyses.*

b. I do not understand the role of the seasonal dummies. If they have the same effects for all utilities, then they cannot have an impact on the relative market shares. Or do these dummies capture the idea that the relative choice of the outside option vs. the inside products depends on season?

*Response: We included the seasonal dummies following an equivalent approach for other logit demand models for SSB by Zheng et al. (2019) and Liu et al. (2016). However, we agree with the reviewer that these* *seasonal dummies have no impact on the specific product’s market share unless the choices of outside vs. inside options are seasonally sensitive (which is worth trying to understand). Thus, we conducted sensitivity analyses with no seasonal dummies and found our model estimates remained robust. We show these estimates as part of S3 Table in the supplemental appendix (S1 File).*

Scenarios

5. Many economists would apply a counterfactual scenario with fiscal neutrality, therefore asking what the tax rates under the UK (or ZA) tax design would be if one wanted to have the same tax revenues (assuming no consumer/producer price reactions) as in the one-MP scenario. Interestingly, this seems to be what the one-MP scenario without reformulation is achieving (this could be mentioned on P22, L381-382).

*Response: We thank the reviewer for this suggestion and agree with him regarding the fiscal neutrality when comparing the current one-MP tax and the one-MP equivalent taxes. Thus, we included the next sentence right after describing the tax revenue across UK and ZA tax designs without reformulation:*

*“Thus, the one-MP equivalent taxes in the absence of reformulation will lead to a scenario of fiscal neutrality (i.e., same tax revenue) compared to the current volumetric one-MP tax.”*

6. In the “sweetness unchanged” reformulation scenario, the authors assume that marginal costs increase by some non-negligible amount. I am not certain that the marginal cost would really be affected by such a reformulation. There would certainly be fixed costs (sourcing, R&D and perhaps marketing to launch the reformulated product). Then, an increase in marginal cost would depend on whether artificial sweeteners are more expensive than sugar-based sweeteners. Can the authors document this point?

*Response: We thank the reviewer for this detailed explanation regarding how reformulation can affect production costs. Unfortunately, we were not able to document that artificial sweeteners are more expensive than sugar-based sweeteners. Thus, we conducted a set of supplementary analyses in which we assumed no differential costs between these two kinds of sweeteners. As expected, purchase and sugar reduction were slightly lower due to lower SSB prices compared to the scenario where we assumed that artificial sweeteners are more expensive than sugar-based sweeteners (see S5 Table in supplemental appendix S1 File). However, results are, in general, similar when assuming or not differential costs between artificial sweeteners and sugar. Additionally, we include the lack of information on the cost of artificial sweeteners as part of our study’s limitations.*

The sweetness unchanged scenario assumes that artificial sweeteners and sugar have similar effects on consumer experience: this is a strong assumption (and the authors implicitly acknowledge that P12, L247-248).

a. In principle, it could be possible to estimate a specific taste parameter for artificial sweeteners. As there are only 30 products, it is not difficult to find how their recipes evolve over time. Accounting for this is likely to affect the results as we know that consumers are attached to certain ‘iconic’ recipes (e.g Coca-Cola regular).

*Response: We thank the reviewer for suggesting this alternative approach that is relevant from a methodological and public health perspective and something we would like to do but are unable to conduct within this data for two reasons. First, we only have nutritional data at the product level for only one time period. Thus, we are unable to see whether recipes evolve over time, which might be unlikely since the volumetric SSB tax in Mexico does not encourage reformulation, as we explained above. Second, the nutritional data only allows us to know whether the product includes artificial sweeteners or not; with no further information on the amount and type of artificial sweeteners (as an aside, the only countries we are aware of that requires declaration of the amounts of each type of artificial sweetener are Chile and Saudi Arabia). Because our reformulation scenarios entail partial reformulation, we might need to know the extent of substitution of sugar for artificial sweeteners that keeps sweetness levels unchanged, which is unfeasible with our current nutritional data.*

b. The analysis of trends in soft-drink markets shows that producers of retailer brands (private labels) and minor national brands are much more likely to reformulate their products than market leaders. The latter tend rather to launch new products with a healthy image (e.g tea-based drinks) or light equivalent to their regular products, or they extend their product portfolio by buying healthier brands. This should be discussed in the Discussion section.
c. There are also some studies that have examined the impact of product dynamics on nutrition - see Griffith, O'Connell et al. (2017), Spiteri and Soler (2018). They show that products entry can sometimes limit the impact of the reformulation of existing products, because consumers tend to stick to their tastes for sugar, fat and salt. This outlines the importance of identifying separately the taste for sugar and the taste for artificial sweeteners.

*Response: Regarding points b and c above, we acknowledge that our model is not flexible enough to model the entry of new products. However, preliminary evidence of the sugar-density tax in South Africa shows that reformulation predominantly drove the sugar drops overpassing the role of the entry of new products. Thus, we updated the paragraph focused on the study’s limitation by including the next sentence:*

*“**Fifth, we did not model product entry as a producer response to the sugar-density tax. However, preliminary evidence of the sugar-density tax in ZA suggests that, compared to reformulation, net product entry (i.e., entry minus exit) plays a minor role in the industry response to the sugar-density tax[51].”*

7. It would be interesting to decompose more formally the effect of the tax + reformulation scenarios into three components: (1) a tax-induced price effect holding composition constant; (2) a change in sugar content at post-tax price level holding post-tax market shares constant; (3) the change in post-tax market shares (at post-tax price levels) that may be induced by the change in demand following reformulation. This third component thus identifies the likely reaction of consumers to reformulation. It is null under the current “sweetness unchanged” scenario but it would not be null if consumers had a specific (dis)taste for artificial sweeteners.

*Response: We thank the reviewer for these suggested analyses. According to our best understanding, our current analyses for the one-MP tax and the international taxes without reformulation are equivalent to scenario (1) above. Moreover, our scenario of “sweetness unchanged” is likely to be close to scenario (2) because we assumed a sugar reduction with no distinguishable flavor change for the consumers, and thus the purchase changes are driven by the post-tax prices and not market share. Regarding scenario (3), as we specified above, we are not able to estimate a specific taste parameter for artificial sweeteners. Thus, we cannot decompose the tax-reformulation effect for the case of “sweetness unchanged”. For the reformulation case of “sweetness reduction”, we already pointed out that the reformulation under the ZA tax design is the main factor behind purchase drops due to the small tax effect on prices. Meanwhile, we might pursue the tax-reformulation decomposition for the UK tax design; however, we anticipate a major role linked to prices compared to reformulation due to the tax effect on prices under reformulation is similar to its counterpart with no reformulation.*

8. A potential drawback of discrete choice models is that the market size is fixed or varies exogenously (Q_t in equations A.2.2 and A.2.1.). This is probably a reasonable assumption in the Mexican context and given that water is the outside option. But this should be discussed somewhere.

*Response: We thank the reviewer for pointing out this limitation as well as the reasonable nature of the related assumption. As a consequence, we included the next sentence when describing our study’s limitations and implications:*

*“Finally, and as standard in logit demand models, we hold fixed the monthly market size (i.e.,* $Q_{t}$ *), under the different tax simulation scenarios (see equations S.1 and S.2 in S1 File). Thus, we assumed no variation in the overall volume sales of non-dairy and non-alcoholic beverages due to the SSB tax. This assumption implies the full substitution of taxed beverages for either untaxed beverages or the outside option, which seems feasible because these latter options mainly correspond to water.”*

9. The instrumental variables only capture shocks on the prices of SSB products. Hence, the estimated marginal utility of income (the price coefficient) will essentially reflect the variations in SSB market shares in response to price shocks. The estimates may underweight the price response of diet products and water. This should be mentioned in the discussion.

   *Response: For the sensitivity analyses, we used products’ prices from other cities as instruments, which impacts both taxed and untaxed products. We found that our demand model and simulation estimates remained robust to the introduction of this price-related instrument. Thus, it seems that our preferred set of instruments (which does not include prices) does not underweight the price response of untaxed products. Thus, we include the next sentence in the section of “Demand model estimates and model fit”:*

*“The estimated structural paraments from the demand model were robust to an increase in the numbers of draws in equation (3), the exclusion of the seasonality dummy variables, and the inclusion of prices from other cities as part of the instrumental variables.”*

Allais, O., F. Etilé and S. Lecocq (2015). "Mandatory labels, taxes and market forces: An empirical evaluation of fat policies." Journal of health economics 43: 27-44.
Berry, S., J. Levinsohn and A. Pakes (1995). "Automobile prices in market equilibrium." Econometrica: Journal of the Econometric Society: 841-890.
Bonnet, C. and V. Réquillart (2013). "Tax incidence with strategic firms in the soft drink market." Journal of Public Economics 106(0): 77-88.
Dubois, P., R. Griffith and M. O'Connell (2020). "How well targeted are soda taxes?" American Economic Review 110(11): 3661-3704.
Etilé, F., S. Lecocq and C. Boizot-Szantai (2020). "Market heterogeneity and the distributional incidence of soft-drink taxes: evidence from France." European Review of Agricultural Economics.
Fox, J. T., K. il Kim, S. P. Ryan and P. Bajari (2012). "The random coefficients logit model is identified." Journal of Econometrics 166(2): 204-212.
Griffith, R., M. O'Connell and K. Smith (2017). "The importance of product reformulation versus consumer choice in improving diet quality." Economica 84(333): 34-53.
Spiteri, M. and L.-G. Soler (2018). "Food reformulation and nutritional quality of food consumption: an analysis based on households panel data in France." European journal of clinical nutrition 72(2): 228-235.

6. PLOS authors have the option to publish the peer review history of their article ([what does this mean?](https://journals.plos.org/plosone/s/editorial-and-peer-review-process#loc-peer-review-history)). If published, this will include your full peer review and any attached files.


If you choose “no”, your identity will remain anonymous but your review may still be made public.


**Do you want your identity to be public for this peer review?** For information about this choice, including consent withdrawal, please see our [Privacy Policy](https://www.plos.org/privacy-policy).

Reviewer #1: **Yes:** Violeta Chacón

Reviewer #2: **Yes:** Fabrice Etilé

1. Nevo A. Mergers with differentiated products: The case of the ready-to-eat cereal industry. RAND J Econ. 2000;31: 395–421. doi:10.2307/2600994
