# Supplementary material for: Simulating international tax designs on sugar-sweetened beverages in Mexico
Source: PLoS One. 2021 Aug 19;16(8):e0253748. doi: 10.1371/journal.pone.0253748 (PMC8375996; doi:10.1371/journal.pone.0253748)
Supplement: Attachment — Submitted filename: Response_reviewer.docx [file pone.0253748.s010.docx]

**Manuscript Number:** PONE-D-20-36090R1

**Manuscript title:** Simulating international tax designs on sugar-sweetened beverages in Mexico

**Response to Reviewers**

*Response: Thank you for being able to review our reviewed version of our manuscript. We found all your suggestions and comments relevant to improve our manuscript. We respond to each in turn below. The major change in the new version of our manuscript corresponds to set the demand model with no seasonal dummies as our preferred model specification, as explained in detail below. As a result of this change, we updated tables and figures from the structural model. In general, our overall findings and conclusions remained similar to our previous submission.*

Reviewer #2: The authors did a great job in dealing with most of my comments. I have only four comments left:
- I am not fully satisfied with the answer to my comment 2b about the estimated standard-deviation of random price coefficient, which is not significant and close to zero: the authors consider that this implies that “households observed characteristics drive price heterogeneity in utility”. This is rather far-fetched, as the choice of working on aggregate market shares and the choice of IVs explain this result. Please reformulate appropriately and add a footnote to mention the identification issue (with the reference to Fox, Kim et al).

*Response: We removed the sentence “households observed characteristics drive price heterogeneity in utility” when describing the findings from our demand model. Then, we include the next sentence based on the suggested reference as follows:*

*“The small and statistically insignificant standard deviation of the random price coefficient might arise from the lack of price variation. Fox et al. (2012) highlight that the identification of the random coefficients relies on the variation in the variables of interest. In our demand model specification, the lack of price variation might result of the use of instruments (i.e., the tax implementation and sugar costs), which impact all SSB and entail a correlation across taxed beverages, and the data aggregation at the urban Mexico level, which overlooks market variations across Mexican cities.”*

- S1 Table, computation of the first-stage F-stat: given the legend of the Table, it is not clear to me that you included the sugar content variable, and the product fixed-effects as controls. Please revise the table and/or the statistics appropriately.

*Response: The first-stage regression includes the sugar content variable and the product fixed-effects as controls. However, we failed in including this description in the S1 Table. Thus, the footnote for the S1 Table now includes the next sentence:*

*“Note: Model includes the sugar content variable and the brands fixed-effects as controls.”*

*Additionally, there is a slight change in the estimates of the first-stage regression because of removing the seasonal dummies from our demand model specification as described below.*

- About the inclusion of seasonal dummies that do not vary across inside options: this is definitely not standard in the empirical IO and in the quantitative marketing literature. Hence, you should better justify their inclusion in your main text: why would we expect specific seasonal shifts in the taste for inside options vs. the outside option?

*Response: Our model was robust to the inclusion/exclusion of the seasonal dummies. We now set the model that does not include these dummies as our preferred model specification. Thus, we re-write equation (1) and related equations by removing the seasonal dummies. Conversely, we now set the demand model with seasonal dummies as part of the sensitivity analyses. Therefore, we updated all relevant tables and figures and their descriptions resulting from the exclusion of the seasonal dummies. Our findings remained similar to our previous model specification.*

- Please provide in the Appendix the table of the marginal costs by brand j that were estimated from the structural supply-side model. This is important for ascertaining the reliability of the model.

*Response: We are contractually not allowed to provide results at the product or brand level following the data use agreements with the data vendor (The Nielsen Company). Given this, we have tried to address the reviewer’s comment by including a graph with the de-identified distribution of marginal costs across products and the average monthly marginal costs by the full categories of taxed and untaxed beverages from 2012 to 2015. Hence, we included the next sentence after describing the results of the demand model.*

*“Based on the demand model estimates and the first-order conditions for the supply model, we recovered the marginal costs. In S2 Fig in the supplemental appendix S1 File, we present the distribution of marginal costs across products and the average marginal costs by the full categories of taxed and untaxed beverages from 2012 to 2015. We found these marginal costs displayed, in general, a stable pattern for the full period of interest.”*
